# $^1$H, $^{13}$C and $^{15}$N assignments of human Grb2 free of ligands

**DOI:** 10.1007/s12104-020-09970-7

**Published:** 2020-08-25

**Authors:** Louise Pinet, Ying-Hui Wang, Anaïs Vogel, Françoise Guerlesquin, Nadine Assrir, Carine van Heijenoort

**Affiliations:** 1 Department of Analytical and Structural Chemistry and Biology, Institut de Chimie des Substances Naturelles, CNRS UPR2301, Université Paris-Saclay, 1, av. de la terrasse, 91190 Gif-sur-Yvette, France; 2 Present Address: Department of Biochemistry, University of Zurich, Winterthurerstrasse 190, 8057 Zurich, Switzerland; 3 Present Address: SGS Taiwan LTD, No.38, Wu Chyuan 7th Rd., New Taipei Industrial Park, Wu Ku District, New Taipei City, 24890 Taiwan; 4 Present Address: NG Biotech, ZI Courbouton, 35480 Guipry, France; 5 LISM, Institut de Microbiologie de la Méditerranée, CNRS and Aix-Marseille University, Marseille, France

**Keywords:** Growth factor receptor-bound 2 (Grb2), Adaptor protein, SH2, SH3, RTK signaling

## Abstract

Growth factor receptor-bound 2 (Grb2) is an important link in the receptor tyrosine kinase signaling cascades. It is involved in crucial processes, both physiological (mainly embryogenesis) and pathological (different types of cancer). Several binding partners of all three domains (SH3–SH2–SH3) of this adaptor protein are well described, such as ErbB family members for the SH2 domain and Sos for the SH3 domains. How the different domains interact with each other, both structurally and functionally, is still unclear. These interactions could be essential for regulation processes, and therefore are of great interest. Although a lot of structural data on Grb2 exist, they describe either individual domains, ligand-bound conformations, or frozen pictures of the protein captured by crystallography. Here we report the assignment of backbone and of $^{13}\hbox {C}_\beta$ chemical shifts of full-length, apo-Grb2 in solution. In addition to the assigned conformation corresponding to three well-folded domains, a set of peaks compatible with the presence of an unfolded conformation of the N-terminal SH3 domain is observed. This assignment paves the way for future studies of inter-domain interactions and dynamics that have to be taken into account when studying the regulation of Grb2 interactions and signaling pathways.

## Biological context

Growth factor receptor-bound 2 (Grb2) is an adaptor protein essential for early steps of embryogenesis, especially differentiation (Cheng et al. 1998), but it was also shown to be upregulated in a subset of breast cancers (Daly et al. 1994). It was originally discovered in humans as the missing link between receptor tyrosine kinases (RTKs) and the Ras/MAPK pathway (Lowenstein et al. 1992). Grb2 is 217 residues long (with a molecular weight of 25 kDa) and is made up of three domains: a central SH2 domain, a well-described type of phosphotyrosine-binding domain, is flanked by two SH3 domains (called NSH3 at the N-terminus and CSH3 at the C-terminus) that bind to proline-rich motifs. Grb2 binds to phosphorylated RTKs via its SH2 domain and to the Son of sevenless guanine nucleotide exchange factor (Sos) via its SH3 domains, triggering Ras/MAPK pathway signaling (Lowenstein et al. 1992; Gale et al. 1993; Buday and Downward 1993), but is also involved in other signaling pathways (Gu and Neel 2003; Ravid et al. 2004; Yamazaki et al. 2002).

The structures of the NSH3 domain in complex with Sos proline-rich peptides (PDB 1GBR) and of the CSH3 free domain (PDB 1GFC) were solved by NMR soon after Grb2 identification (Goudreau et al. 1994; Wittekind et al. 1994; Kohda et al. 1994). The SH2 domain structure was solved shortly after by X-ray crystallography (PDB1GHU) (Thornton et al. 1996). In the meantime, the 3.1 Å resolution structure of full-length Grb2 was also solved by X-ray crystallography (PDB 1GRI) (Maignan et al. 1995). In this structure Grb2 is a dimer, but the oligomeric state of Grb2 in solution remains largely controversial, just like the presence and nature of inter-domain interactions. The only detailed analysis of full-length Grb2 in solution was performed by NMR by Yuzawa et al. (2001), but with both SH2 and SH3 ligand peptides present. The behavior of apo-Grb2 in solution is therefore still poorly defined, although it is essential to decipher the interdependence of its domains and of their binding to different motifs.

## Methods and experiments

### Sample preparation

The gene coding for full-length Grb2 (synthetized by MWG) was inserted into a pETM11 vector also containing the gene conferring resistance to kanamycin. The construct contains a N-terminal His6 tag followed by a TEV protease cleavage site for protein purification. Grb2 plasmid was transformed into BL21 star(DE3) *E. coli* cells. A colony from the transformation was seeded into 2xYT medium, and bacteria were then successively grown at 37 °C and diluted into unlabeled M9 minimal medium, $^{15}$N-$^{13}$C M9, and finally $^{15}$N-$^{13}$C deuterated M9, for 80% deuteration of the final sample. In the M9 media, $\hbox {NH}_4$Cl concentration was 1 g/L independently of its isotopic labeling, while glucose concentration was 4 or 3 g/L for unlabeled or $^{13}$C labeled glucose, respectively. Induction with isopropyl $\beta$-D-1-thiogalactopyranoside (IPTG) was performed and bacteria were harvested after overnight protein expression at 25°C.

Cell lysis was obtained by disruption (Constant Systems Ltd.), the lysate was ultracentrifuged and the supernatant filtered before loading on a HisTrap FF crude column (GE Healthcare Life Sciences). After elution, the His6 tag was removed by TEV cleavage, leaving two additional residues G and A at the N-terminus compared to the native Grb2 sequence. The final protein product therefore had 219 residues. Excess of TEV protease, free tag and uncleaved protein was removed with a Ni-NTA resin (Thermo Scientific), and purification was completed with gel filtration on a Superdex-75 column (GE Healthcare Life Sciences). Despite deuteration during expression and given that the purification was performed in $^1\hbox {H}_2$O, amide groups were all back-exchanged from $^2$ to $^1$H before NMR measurements without need for any additional step. The final yield of [U-$^{15}N$; U-$^{13}C$; U-80% $^2H$] Grb2 production was about 40 mg/L culture. 200–400 $\upmu$M NMR samples were prepared in PIPES buffer at pH 7.2 (40 mM PIPES, 150 mM NaCl, 2 mM TCEP).

### NMR spectroscopy

Spectra were recorded at 308K with Bruker Avance NMR spectrometers of proton frequencies 600, 800 and 950 MHz equipped with TCI cryoprobes. Backbone and $^{13}\hbox {C}_\beta$ assignments were obtained using a common combination of 3D experiments: HNCO, HN(CA)CO, HNCA, HNCACB and HN(CO)CACB. Assignments of Grb2 isolated SH2, NSH3 and CSH3 domains were also obtained and were used to clear up a few ambiguities in the assignment of the full-length protein. The data were processed using Topspin3.5 (Bruker) and analyzed using CCPNMR (Vranken et al. 2005).

### Secondary structure verification

Overall structural integrity of each domain (SH2, CSH3 and the main form of the NSH3 domain) was assessed using chemical shifts and TALOS+ (Shen et al. 2009), and compared with secondary structures in Grb2 crystal structure (PDB 1GRI).

## Extent of assignments and data deposition

**Fig. 1:**
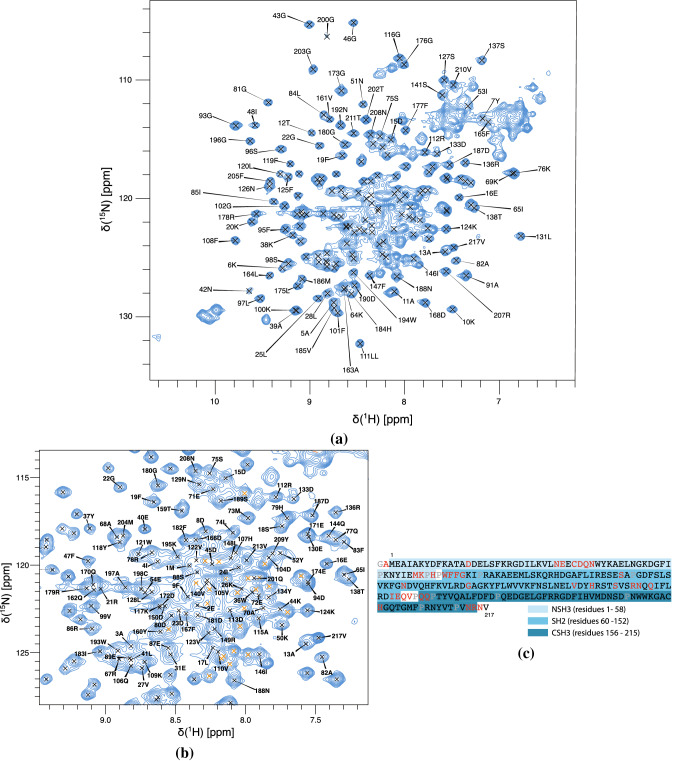
Assignment of backbone $^1$H-$^{15}$N pairs of full-length Grb2. **a**)Assigned $^1$H-$^{15}$N HSQC spectrum of [U-$^{15}$N; U-$^{13}$C; U-80% $^2$H]-Grb2 (308 K, PIPES buffer pH 7.2, 600 MHz), with a zoom on the central region in (**b**). Some of the peaks believed to correspond to the unfolded form of the NSH3 domain are marked in orange. **c** Sequence of the construct of Grb2 that we used, corresponding to Uniprot accession number P62993 with two additional residues at the N-terminus. The missing assignments are shown in red along the sequence, while grey residues are prolines and the N-terminal residue. The limits of the three domains as defined by ProRule are given

*Assignment of backbone* $^{15}$*N-*$^1$*H*  Resonance assignment of full-length Grb2 has been obtained using a series of heteronuclear experiments. The backbone sequential assignment of the $^1$H-$^{15}$N HSQC spectrum is presented in Fig. 1. The unassigned residues mainly belong to the unstructured edges of the three domains, or to the linkers between them, which indicate specific dynamic features of these regions. Additionally, we could not assign the region around cysteine 32, which is located in a loop that was shown to exhibit intermediate exchange in the context of the isolated NSH3 domain by NMR (Goudreau et al. 1994) and where electron density is missing in full-length Grb2 crystal structure (PDB 1GRI). Finally, a few isolated residues could not be assigned (0A, 14D, 90S, 103N, 114G, 132V, 135H, 199H).

Overall, 173 backbone $^{15}$N-$^1$H resonances were assigned (84% of the residues excluding prolines and the N-terminal residue, or 79% of all residues).

*Carbon assignment*  HNCO and HN(CA)CO spectra allowed assignment of 86% of all $^{13}$C’ carbons, and HNCA, HNCACB and HN(CO)CACB spectra allowed assignment of 88% of all $^{13}\hbox {C}_\alpha$ and 86% of all $^{13}\hbox {C}_\beta$ resonances, yielding good coverage of the whole sequence.

*Additional peaks in the spectra*  The isolated NSH3 domain of Grb2 has been shown to exchange between a folded and an unfolded conformation under acidic conditions (Goudreau et al. 1994), while its ortholog in *Drosophila* (Drk) has been shown to undergo a similar equilibrium in physiological buffer (Zhang et al. 1994).

In our spectra of Grb2 at neutral pH (7.2), a few tens of peaks are present that we excluded as belonging to any unassigned residue. They are mainly located in the central part of the spectrum in the proton dimension, suggesting that they are part of an unfolded conformation. Moreover, some of these peaks could be assigned to small segments consistent with parts of the NSH3 domain sequence (assignment not reported here). Our hypothesis is that these additional peaks correspond to an unfolded form of the NSH3 domain in full-length Grb2, the other two domains being only present in their fully folded form. From peak volumes, the unfolded to folded molar ratio is of the order of 1:1.

*Data deposition* The $^1$H, $^{13}$C and $^{15}$N chemical shifts have been deposited in the BioMagResBank (http://www.bmrb.wisc.edu/) under the BMRB accession number 50082.

## Data Availability

The presented assignments were deposited to the BMRB under the entry 50082.
